# Evaluation of keratinized gingiva changes with buccal-based modified palatal flap in anterior maxillary implants

**DOI:** 10.34172/joddd.2022.031

**Published:** 2022-11-15

**Authors:** Mahdi Sadeghi, Mohamad Ali Ghavimi, Reza Khorshidi, Faeze Moini, Arezou Ghoreishizadeh

**Affiliations:** ^1^Department of Oral and Maxillofacial Surgery, Faculty of Dentistry, Tabriz University of Medical Sciences, Tabriz, Iran; ^2^Department of Periodontology, Faculty of Dentistry, Ardabil University of Medical Sciences, Ardabil, Iran; ^3^Department of Pediatric Dentistry, Faculty of Dentistry, Tabriz University of Medical Science, Tabriz, Iran

**Keywords:** Dental implant, Gingiva, Peri-implantitis

## Abstract

**Background.** Studies have shown a significant relationship between the width and thickness of keratinized gingiva around the implant and changes in marginal bone level, with a significant effect on the health and esthetic of tissues around the implant, especially in the anterior region of the maxilla, which is an esthetic area.

**Methods.** Ten patients referring to the Faculty of Dentistry seeking implant placement in the anterior maxilla were included in the study. The connective tissue of the palatal gingiva of the surgical site was folded to the buccal aspect with the buccal base, and the thickness and width of keratinized gingiva around the buccal surface of each implant were measured in three time intervals, including before surgery and 6 and 12 weeks after surgery. Based on the results of the Kolmogorov-Smirnov test, Friedman test and repeated-measures ANOVA were used to analyze the data.

**Results.** The intervention significantly affected changes in the gingival thickness. After the intervention, gingival thickness significantly increased compared to the baseline (*P*<0.05). The results also showed that the intervention did not significantly affect the width of keratinized gingiva. The width of keratinized gingiva at baseline was not significantly different from the two time intervals after intervention (*P*>0.05).

**Conclusion.** Buccal-based modified palatal flap in anterior maxillary implants increased the thickness of keratinized gingiva, with no significant effect on the keratinized gingiva width.

## Introduction

 The importance and necessity of keratinized gingival width around the implant have been discussed for more than 20 years, and its appropriate value has been reported as>2 mm in various studies.^1,2^ Studies have shown that the lack of proper keratinized gingiva increases plaque formation, gingivitis, bleeding on probing, and gingival resorption around the implant.^3^ In addition, bone resorption around the implant with wider keratinized gingiva is less common, and in general, the relationship between the health of the tissues around the implant and the width of the keratinized gingiva has been reported to be statistically significant.^3^ The thickness of keratinized gingiva is another important parameter of implant health, and low keratinized gingiva thickness around teeth is known as thin biotype gingiva, inherently increasing the risk of gingival resorption following restorative, surgical, or mechanical traumas. A similar mechanism can be present in the tissues around implants. Individuals with thin gingival biotypes around the teeth also have less gingival thickness around the implants, making them more susceptible to gingival resorption.^4^ Nowadays, with the introduction of implants with rough surfaces and the increased risk of peri-implantitis in these implants, the quality of keratinized gingiva has become more important.^5^ In general, it can be concluded that actions that increase the width and thickness of the keratinized gingiva around the implant will increase the stability of the implant.^6^ Various surgical methods have been introduced to augment keratinized gingiva, which in addition to the above, will lead to better oral hygiene due to reducing pain and discomfort when brushing and increasing esthetic.^5^ One of these methods is the use of the soft tissue autograft technique with free gingival transplantation or free connective tissue transplantation (FCTG), both of which increase the width of keratinized gingiva equally. A study showed that although FCTG increases the implant’s stability, it does not have acceptable esthetic outcomes due to excessive contraction.^7^ In addition, this method requires a transplant donor site, which increases the patient’s recovery time and discomfort.^8^ In this method, the possibility of necrosis and contraction of the graft increases due to the separation of the connective tissue from the blood bed.^9^ Nevertheless, this method is still considered the gold standard for reconstructing soft tissue around teeth and implants.^10^ An alternative surgical method in this study to improve the quality of keratinized gingiva in anterior maxillary implants is to use a rotational flap of palatal connective tissue to the buccal region. One of its advantages is that the connective tissue does not separate from its base and only rotates from the palatal to the buccal aspect. In addition, the donor site is located at the recipient site, eliminating problems with the donor site, such as postoperative pain, the possibility of infection, and so on. Therefore, the patient is relieved of the next additional surgical step and pain in the transplant donor area; in addition, the patient benefits from all the advantages mentioned in the FCTG method.

## Methods

 To determine the sample size, we used the results of a study by Cardaropoli et al.^11^ Considering the average keratinized gingiva before and 12 months after surgery, i.e., 23.2 ± 0.56 and 3.45 ± 0.85, respectively, an error rate of 0.05, and a test power of 90%, seven samples were obtained. To increase the study’s validity, 30% was added to the sample size, which was finally considered to 10 samples. Then the patients in the Faculty of Dentistry, who needed the implant treatment in the anterior maxilla and met the inclusion criteria, were included in the study.

###  Inclusion criteria

1. Candidate for implant placement in the central and lateral teeth area
2. The minimum width of the bone was 4.5 mm
3. The minimum height of the bone was 12 mm
4.  >6 months since tooth extraction.

###  Exclusion criteria

1. Uncontrolled periodontal disease
2. Presence of teeth in the implant area
3. Uncontrolled systemic disease
4. History of smoking in the last 6 months
5. Pregnant and lactating women
6. Patients with connective tissue problems
7. Radiotherapy and chemotherapy
8. Initial stability<30 N.cm so that there was no indication for single-stage implant surgery.

**Figure 1:**
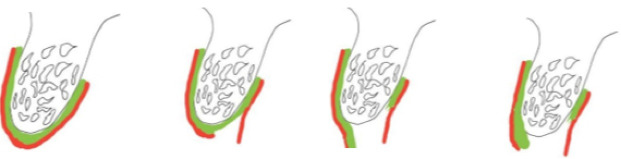
The surgical technique.

Ten dental patients underwent surgery by a maxillofacial surgeon with 5 years of specialized work. Standard surgeries were performed, and in all the patients, a fixture with a suitable diameter was used according to the available bone and the DIO dental implant system. A split-thickness crestal incision was made at the top of the crest, which was 0.5 mm towards the palate. Then supraperiosteal dissection was performed on the palatal side up to 2 cm. At the end of the palatal dissection, a periosteal incision was made, and the periosteum and connective tissue were folded to the buccal side and sutured by absorbable sutures (Figure 1). Then, the fixture was placed as standard so that the initial torque was>30 N.cm. It should be noted that if the torque was lower than this value, the patient was excluded from the study, and the surgery was performed in two stages. Then, the healing abutment was attached with a diameter of 4.5, a cuff of 4, and a height of 4.5 mm (one-step surgical procedure).^12^

 Baseline data were measured as follows: Before surgery, three points were marked with a marker from the buccal aspect, and the thickness of keratinized gingiva at these three points and in the mid-buccal area at different heights was measured using a #25 endodontic spreader. Then the width of keratinized gingiva was measured by a gauge at three points: mesial, mid-buccal, and distal from the mucogingival line to the free gingival margin. After preparing the surgical site, placing the fixture, and connecting the healing abutment, the surgeon also measured the marked points from the top of the healing abutment to repeat the measurement from the same points the next time. After surgery, the surgical site was sutured with 4-0 Vicryl sutures. Finally, the width and thickness of keratinized gingiva were measured preoperatively and at 6- and 12-week intervals and compared.

###  Statistical analysis

 In this study, 10 patients were examined. This study was performed in three time intervals: before the intervention and 6 and 12 weeks after it. Based on the results of the Kolmogorov-Smirnov test, for the analysis of variables whose significance level was<0.05, nonparametric tests and Friedman statistical test were used. Also, parametric analytical statistics and repeated-measures ANOVA were used.

## Results

Table 1 presents the results of the study regarding the average thickness of gingival tissues at three points and at different intervals. The results showed that the intervention had a significant effect on changing the mean gingival thickness. In other words, the mean gingival thickness increased significantly after the intervention (*P*<0.05).

**Table 1:** Comparison of gingival thickness at three time intervals

**Gingival thickness: mean (standard deviation)**			**The significance level ** ^*^		
Baseline (B)	6 weeks later (6 wk)	12 weeks later (12 wk)	B & 6 wk	B and 12 wk	6 wk and 12 wk
1.86 (0.58)	2.5 (0.77)	3.07 (1.09)	>0.001	>0.001	0.007

*Significance level at the three time intervals (from the results of the repeated-measures test).

Table 2 presents the results of the study of gingival width at three points and at different time intervals. The results showed that the intervention had no significant effect on changing the gingival width. In other words, the total gingival width before intervention was not significantly different from that after intervention (*P*>0.05).

**Table 2:** Comparison of gingival width (overall) at the three time intervals

**Gingival width (overall) Middle (first quarter-third quarter)**			**The significance level ** ^*^			**The significance level** ^**^
Baseline (B)	6 weeks later (6 wk)	12 weeks later (12 wk)	B and 6 wk	B and 12 wk	6 wk and 12 wk^***^	B, 6 wk, and 12 wk
5.41 (4.3–7.54)	5.83 (5.12–7.08)	6.41 (5.54–7.3)	0.759	0.779	0.131	0.368

**Significance level at the three time intervals (from the results of the Friedman test). *Significant level between the results of two time intervals (from the results of Wilcoxon's test). ^***^Significance level between the results of two time intervals (from the results of paired *t *test).

## Discussion

 The present study aimed to evaluate the changes in keratinized gingiva using a modified palatal flap with a buccal base in anterior maxillary implants. A 2020 study by Kabir et al^13^ showed that reduced keratinized gingival width is a risk factor for the severity of peri-implant mucositis, and keratinized gingiva>2 mm reduces this risk.

 Grischke et al^14^ showed a significant relationship between reduced keratinized gingiva and the severity of peri-implant mucositis in patients at low risk of periodontal disease with no previous or present history of periodontitis. The Gharpure et al study showed that although keratinized gingiva is important for implant success, few studies have evaluated the soft tissue graft around the implant, with most studies focusing on the bone around the implant.^15^ Therefore, according to the data obtained from various studies, studies such as the present study are important to improve the quality and quantity of soft tissue. Kan et al^16^ used connective tissue gingival grafting (CTG) with immediate implant placement, reporting that although interproximal area resorption after implantation is not common, it is common in facial gingiva and the severity of resorption. In thin gingival biotypes, CTG improves the gingival phenotype and turns thin gingiva into thick ones.

 Tavelli et al^17^ attributed the induction of keratinization of the superficial epithelium in a natural dentition to the underlying connective tissue. Nevertheless, this may not be due to differences in the anatomy of periodontal tissues around the implant in the case of implants. However, the possibility of connective tissue influencing the keratinization of the superficial epithelium and improving its quality remains strong. In addition, studies like the one above can show the importance of retaining and benefiting from the connective tissue of the surgical site, as performed in the present study.

 A meta-analysis by Tavelli et al^17^ showed that bi-laminar techniques resulted in the greatest increase in gingival thickness. The surgical method in the present study is a subset of bi-laminar methods; according to the results and its significance in increasing the thickness of the gingiva, this study is consistent with the above studies. Chung et al^18^ introduced CTG as an effective technique, reported two cases of necrosis, and introduced this technique as a method with high technical sensitivity. The present surgical method, while having all the advantages of CTG and its disadvantages, such as the lack of a suitable blood substrate, does not require a donor area and is not technique-sensitive. Therefore, it can be considered as one of the best methods.

 Another issue is the time of grafting or other corrective surgeries. Tavelli et al^17^ showed that the treatment outcome is not affected by the time of grafting, i.e., grafting has the same results when the implant is placed or uncovered. Therefore, according to the results of the studies mentioned above, corrective surgeries can be performed simultaneously with the placement of dental implants, and considering the advantage of no need for a second surgery, this issue can be considered one of the strengths of the present study.

 In the present study, although the thickness of keratinized gingiva increased significantly, the width of keratinized gingiva did not increase significantly, consistent with a systematic review by Tavelli et al.^17^ Apically positioned flap leads to an increase in keratinized gingival width. Therefore, it can be suggested that the present method should be combined with apically positioned flap in future studies.

## Conclusion

 Buccal-based modified palatal flap in anterior maxillary implants increased the thickness of keratinized gingiva, with no significant effect on the width of keratinized gingiva.

## Acknowledgments

 None.

## Author Contributions

 MG and MS were mainly responsible for the design and supervision of the study. RK and AG were involved in the data entering and performed the analysis. FM papered the manuscript. All the authors revised and approved the manuscript.

## Funding

 This study was part of an approved study at Tabriz University of Medical Sciences (grant number: IR.TBZMED.REC.1400.023).

## Ethics Approval

 This study was part of an approved study by the Research Ethics Committee of the Tabriz University of Medical Sciences (IR.TBZMED.REC.1400.023).

## Competing Interests

 None.
